# Klokwerk + study protocol: An observational study to the effects of night–shift work on body weight and infection susceptibility and the mechanisms underlying these health effects

**DOI:** 10.1186/s12889-016-3317-1

**Published:** 2016-08-02

**Authors:** Bette Loef, Debbie van Baarle, Allard J. van der Beek, Linda W. van Kerkhof, Daniëlla van de Langenberg, Karin I. Proper

**Affiliations:** 1 Center for Nutrition, Prevention and Health Services, National Institute for Public Health and the Environment, P.O. Box 1, 3720 BA Bilthoven, The Netherlands; 2 Department of Public and Occupational Health, EMGO Institute for Health and Care Research, VU University Medical Center Amsterdam, Amsterdam, The Netherlands; 3 Center for Infectious Disease Control, National Institute for Public Health and the Environment, Bilthoven, The Netherlands; 4 Center for Health Protection, National Institute for Public Health and the Environment, Bilthoven, The Netherlands; 5 Institute for Risk Assessment Sciences, Utrecht University, Utrecht, The Netherlands

**Keywords:** Night–shift work, Body weight, Infection susceptibility, Sleep, Physical activity, Diet, Light exposure, Vitamin D, Immunological factors

## Abstract

**Background:**

Night–shift work may cause severe disturbances in the worker’s circadian rhythm, which has been associated with the onset of health problems and diseases. As a substantial part of the workforce is exposed to night–shift work, harmful aspects of night–shift work should not be overlooked. The aim of the Klokwerk + study is to study the effects of night–shift work on body weight and infection susceptibility and the mechanisms underlying these health effects. First, we will study the relation between night–shift work exposure and body weight and between night–shift work exposure and infection susceptibility. Second, we will examine the mechanisms linking night–shift work exposure to body weight and infection susceptibility, with a specific focus on sleep, physical activity, diet, light exposure, vitamin D level, and immunological factors. Lastly, we will focus on the identification of biomarkers for chronic circadian disturbance associated with night–shift work.

**Methods/design:**

The design of this study is a prospective observational cohort study consisting of 1,960 health care workers aged 18–65 years. The study population will consist of a group of night–shift workers and an equally sized group of non–night–shift workers. During the study, there will be two measurement periods. As one of the main outcomes of this study is infection susceptibility, the measurement periods will take place at approximately the first (September/October) (T0) and the last month (April/May) (T1, after 6 months) of the flu season. The measurements will consist of questionnaires, anthropometric measurements, a smartphone application to determine infection susceptibility, food diaries, actigraphy, light sensors, and blood sample analyses.

**Discussion:**

The Klokwerk + study will contribute to the current need for high–quality data on the health effects of night–shift work and its underlying behavioral and physiological mechanisms. The findings can be the starting point for the development of interventions that prevent negative health effects caused by night–shift work. In addition, the identification of biomarkers indicative of loss of homeostasis due to circadian disturbance may be an important asset in monitoring the effects of such interventions.

## Background

In modern society, our economy operates 24/7 with the principles of supply and demand going on at all times. Consequently, a substantial part of the workforce is required to work outside the regular 9 to 5 office hours, with approximately one in five European workers being exposed to schedules that include night shifts [1]. Engaging in shift work, and particularly in night–shift work, may lead to the disturbance of workers’ natural circadian rhythm of biological functions that may subsequently interfere with their health and well–being [2]. The Klokwerk consortium was formed to assess the potential adverse health effects of night–shift work. Within the consortium two studies are conducted. The Klokwerk study (study protocol described elsewhere [3]) implements a comprehensive protocol that has been developed to conduct detailed assessment of exposure to the multi–dimensional aspects of night–shift work. The second aim of the Klokwerk study is the identification of long–term markers of circadian disruption. The Klokwerk + study is described here. While the two studies both have a unique aim, they are overlapping in the methods that are applied. Therefore, combining data from the two will provide unique insights in the adverse health effects of night–shift work, beyond what could have been achieved in each study separately.

Besides acute effects, such as sleep disturbances and social problems, night–shift work has also been linked to chronic effects, such as cardiovascular diseases and cancer [4–6]. In addition, evidence is accumulating on the relation between night–shift work and two other major public health problems for today’s society: overweight and infectious diseases [7–9]. Previous studies in mice have found a causal relationship between circadian disturbance and body weight gain [10, 11]. In humans, epidemiological studies have also indicated that overweight and obesity may be more prevalent in night–shift workers compared to non–night–shift workers [9, 12–14]. Besides body weight gain, night–shift work may also cause increased infection susceptibility [7, 15]. Circadian disturbance might increase the risk of becoming infected with an infectious pathogen as well as intensify the severity of an infectious disease once infected. Although multiple (review) studies have found support for the relation between night–shift work and body weight gain [8, 9, 16], and night–shift work and infection susceptibility [7, 15, 17], there is a need for more high–quality studies (i.e. studies of high methodological quality and with a longitudinal design) on this topic in order to draw more convincing conclusions and to examine underlying mechanisms.

The circadian disturbance caused by exposure to night–shift work has been proposed as the driver of multiple pathways that induce these adverse health effects [16, 18, 19]. These pathways can be roughly divided into the following three groups of factors: psychosocial, behavioral, and physiological factors [16, 18–22]. With respect to psychosocial factors, night–shift work may be associated with higher job strain, lower job satisfaction and disturbances in work–life balance [23, 24]. This may induce high levels of stress and consequently contribute to an increase in body weight and infection susceptibility [7, 16, 19, 25]. Secondly, disturbances in day–night rhythm experienced by night–shift workers may bring about behavioral changes in sleep and lifestyle. Besides the irregular sleeping pattern caused by shift schedules [26, 27], night–shift work may also alter sleep quantity and quality [21, 23, 28]. Furthermore, previous studies have indicated that night–shift workers engage in poorer diet behaviors and less physical activity [8, 9, 29, 30], smoke more and consume more alcohol [2, 20, 31]. These behavioral changes may increase night–shift workers’ risk of obesity [16, 32, 33], and may weaken their immune system, making them more susceptible to infection [7, 34–40]. With respect to physiological factors, artificial light exposure and food intake during normal sleeping periods may further disturb the circadian cycle and a lack of sun light exposure may result in an altered vitamin D level [41, 42], which may increase susceptibility to infections and contribute to body weight gain [43–46]. Besides the pathway via vitamin D, circadian disturbance may also have a direct effect on immunological factors by affecting the cellular immune response [17, 47].

Insight into the mechanistic factors underlying the adverse health effects of night–shift work is needed to develop preventive strategies. The use of biological markers may provide an opportunity to determine the presence of chronic circadian disturbance and to monitor the effects of interventions on circadian disturbance long before adverse health effects manifest [48]. Currently used biomarkers, such as melatonin and cortisol [49], have disadvantages: firstly, as these biomarkers are under circadian control, multiple measurements around the clock are required to validate these markers, and secondly, they provide information on acute circadian disturbance, but not on cumulative, chronic circadian disturbance [48]. Therefore, it would be desirable to identify biomarkers that are indicative of loss of homeostasis due to chronic circadian disturbance.

The main aim of this study is to examine the effects of night–shift work on body weight and infection susceptibility and the mechanisms underlying these health effects. First, we will study the relation between night–shift work exposure and body weight and between night–shift work exposure and infection susceptibility. Second, we will examine the mechanisms linking night–shift work exposure to body weight and infection susceptibility, with a specific focus on sleep, physical activity, diet, light exposure, vitamin D level, and immunological factors. Lastly, we will focus on the identification of biomarkers for circadian disturbance associated with night–shift work.

## Methods/design

### Study design

The design of this study will be a prospective observational cohort study consisting of 1,960 health care workers (both night–shift workers and non–night–shift workers). During the study, there will be two measurement periods. As one of the main outcomes of this study is infection susceptibility, the measurement periods will take place at approximately the first (September/October) (T0) and the last month (April/May) (T1, after 6 months) of the flu season in order to detect sufficient cases of influenza–like illness (ILI) or acute respiratory infection (ARI) [50].

The measurements will consist of questionnaires, anthropometric measurements (i.e. body height, body weight, and waist circumference), a smartphone application to determine infection susceptibility, food diaries, actigraphy, light sensors, and blood samples. At baseline, participants will receive the smartphone application, actigraphy devices, light sensor, and food diary. Furthermore, participants’ height, weight, and waist circumference will be measured and they will be asked to fill in the questionnaire online. The smartphone application will be used to report the presence of ILI/ARI on a daily basis during 6 months (until the second measurement period). The actigraphy devices and light sensor will be worn for 7 consecutive days. The food diary will be kept for 3 consecutive days. At 6 months, the second measurement period will take place, in which the questionnaire, anthropometric measurements, actigraphy, and light sensor measurements will be repeated. Furthermore, the total number of ILI/ARI cases of the past flu season will be determined. Based on an expected incidence of ILI/ARI cases from previous years, it is expected that 175 health care workers will report ILI/ARI (10 %). From these 175 expected cases and from 70 non–night–shift working matched controls (e.g. gender, age), blood samples will be drawn for immunological analyses.

Table 1 shows an overview of the measurement schedule.

**Table 1:** Overview of the measurement schedule

*Measurement methods*	n	Measurement period I (Sept/Oct)	Sept/Oct–Apr/May	Measurement period II (Apr/May)
Questionnaire	1960	One time	−	One time
Anthropometry	1960	One time	−	One time
Smartphone application	1960	Daily	Daily	−
Food diary	1960	3 days	−	−
Actigraphy	260	7 days	−	7 days
Light sensor	260	7 days	−	7 days
Blood sample	245	−	−	One time

### Study population

The study population will consist of 1,960 health care workers aged 18–65 years. In this study, nurses, physicians, and other (allied) health professionals (e.g. physiotherapists, midwifes, dietitians, psychologists) working in a hospital will be included. The study population will consist of a group of night–shift workers and an equally sized group of non–night–shift workers. Health care workers will be allocated to the group of night–shift workers if they work night shifts (shifts between midnight and 06.00 a.m.) for at least 1 night per month over the past 6 months [51]. The non–night–shift work group will consist of health care workers who have not worked night shifts for at least 6 months. Furthermore, different cut–off points will be used to compare night–shift workers and non–night–shift workers based on information on relevant night–shift work aspects, such as number of years of night–shift work and frequency of night–shift work. Besides being 18–65 years and working as a health care worker in a participating hospital, another inclusion criterion is that the participant is expected to be employed as a health care worker during the complete follow–up period.

**Fig. 1:**
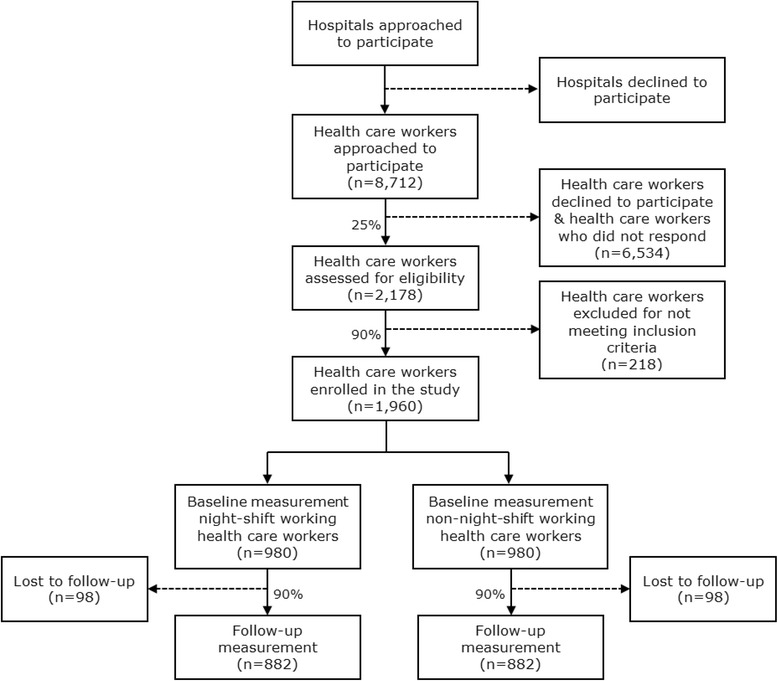
Flow diagram of the recruitment and study procedures and the expected response

The source population of this study will be drawn from several hospitals. A number of large hospitals in The Netherlands will be approached to participate in the Klokwerk + study. After approval of the board, managers, and the works council, the health care workers working in the participating hospitals will be invited to participate by means of an information letter and reply form, which will be sent to them by e–mail or another internal communication system of the hospital. Those willing to participate will sign an informed consent form. In the participating hospitals, the measurements will take place in meetings lasting about an hour. Figure 1 shows the flow diagram of the recruitment and study procedures and the expected response.

### Sample size calculation

The number of participants required for this study was determined based on infection susceptibility, measured by the occurrence of ILI/ARI cases. According to the World Health Organization (WHO), every year, approximately 5–15 % of the population becomes infected with influenza during flu season [52]. Based on the incidence of influenza cases in previous years (Van Beek et al., submitted for publication) and because the incidence of ILI/ARI cases is higher than the incidence of influenza cases, it is expected that approximately 10 % of the study population will develop ILI/ARI. It is hypothesized that night–shift working health care workers will be more susceptible to ILI/ARI than non–night–shift working health care workers. Based on an assumed relative risk of 1.5 to be a relevant difference between the two groups, the expected proportion of ILI/ARI cases is set at 12 % in the group of night–shift workers and at 8 % in the group of non–night–shift workers. With a significance level of 5 % and a power of 80 %, the sample size per group then becomes 882. After including an expected drop–out rate of 10 %, 980 participants per group are needed. Thus, in total, 1,960 health care workers are needed in the study. We expect a response rate of 25 % and we expect that of those responding, approximately 90 % will meet the inclusion criteria. Hence, in total, 8,712 health care workers need to be invited to participate (Fig. 1).

### Study parameters

#### (Night-) shift work

The current study aims to capture all major domains of shift work that have been identified by the international consensus report by Stevens et al. (2011) [53]. Based on this consensus report, the Nightingale study, a cohort study among 60,000 night–shift working and non–night–shift working nurses, already formulated questions regarding all shift work domains (i.e. shift system, cumulative exposure, shift intensity) [51]. In the current study, similar questions will be used in which participants will be asked to report their current work schedule and answer questions about their (night-) shift work history (e.g. number of years of shift work, number of shifts per month) [51].

#### Body weight

Body height, body weight, and waist circumference will be measured by direct measurements executed by the researcher/research assistants. Body Mass Index (BMI) can be calculated by dividing weight in kilograms by the square of height in meters. In addition, the change in BMI after follow–up relative to BMI at baseline can be measured as an indication of potential body weight gain.

#### Infection susceptibility

Infection susceptibility is defined as the development of ILI/ARI. Based on the ILI/ARI definitions of the European Center for Disease Prevention and Control (ECDC) [54], the following symptoms will be taken into account in this study: cough, sore throat, shortness of breath, runny/stuffy nose, fever, feverishness, hoarseness, coughing up mucus, sneezing, and wheezing. An ILI/ARI case will be defined as having two or more of these symptoms (except for sneezing and wheezing) on the same day or as having at least one of these symptoms (except for sneezing and wheezing) during two subsequent days. A mobile phone application has been developed by the University Medical Center Utrecht (UMCU), Julius Center to detect parent–reported ILI cases in children and appeared successful. For the purpose of this study, this app will be further adjusted to make it applicable for the measurement of ILI/ARI in adults. Besides measuring the presence of ILI/ARI, the app will also provide insight into the duration of an ILI/ARI episode. In the app, participants will keep a daily log, in which they can report their ILI/ARI symptoms by selecting their symptoms from a list consisting of the aforementioned symptoms or they can select the box for no symptoms/not more than usual. Participants with an ILI/ARI will be asked to report on a 4-point Likert scale (ranging from not at all to a lot) to what extent the ILI/ARI symptoms bothered them. After an ILI/ARI has occurred, participants will be marked as “recovered” from their ILI/ARI if they report no symptoms for at least two subsequent days or if only one and the same symptom is being reported during a period of 5 days. Recovered participants will receive a concluding questionnaire with questions about ILI/ARI symptoms experienced by other people in their household, sickness absenteeism, presenteeism, other restrictions in daily activities, seeing a doctor, hospital admission, and use of medication. The use of a mobile phone application has appeared to be an easy and efficient way to measure infection susceptibility, resulting in high compliance.

#### Sleep factors

In this study, subjective sleep parameters will be monitored using the Medical Outcomes Study (MOS) Sleep Scale [55]. This questionnaire consists of 12 items that cover the following 6 domains: sleep quantity, sleep adequacy, sleep disturbance, somnolence, snoring, and shortness of breath or headache. The questions relate to the participant’s usual sleep habits during the past 4 weeks. To examine sleep quantity, participants will be asked to report how many hours of sleep they got per day during the past 4 weeks. Besides this question about duration of sleep, participants will be asked to report how long it has usually taken them to fall asleep. In the other 10 items, participants will be asked to indicate on a 6-point Likert scale (ranging from all of the time to none of the time) how often they experienced certain problems related to their sleep. To measure sleep quality, an overall score of multiple domains of the MOS Sleep Scale (9 items) can be calculated. The MOS Sleep Scale showed good validity and reliability [55, 56]. In addition to the MOS Sleep Scale, participants will be asked to indicate on a 5-point Likert scale (ranging from very good to very bad) how they rate their overall sleep quality. Furthermore, in their food and actigraphy diary, participants will report their sleep times and a subsample of participants will wear actigraphy devices (see below). This information will also provide insight into participants’ sleep quantity and quality.

#### Physical activity

Physical activity will be measured using the Short QUestionnaire to ASses Health enhancing physical activity (SQUASH) [57]. In this questionnaire, the duration, frequency, and intensity of leisure time activities, household activities, activity at work and school, and commuting activities during a regular week in the past month are assessed. SQUASH has been found to be a fairly reliable (*r* = 0.58) and reasonably valid (*r* = 0.45) questionnaire to measure physical activity [57]. Furthermore, in a subsample of the study population, physical activity will also be measured objectively using actigraphy devices (GT3X+/GT3XP–BTLE accelerometer, ActiGraph, Pensacola, FL, USA). This subsample will be randomly drawn from the total study population of night–shift workers and non–night–shift workers and will consist of 130 night–shift workers and 130 non–night–shift workers. Participants will wear the actigraphy devices for 7 consecutive days [58]. Participants will keep a short diary on the exact wearing times of the devices, the date, sleep times, time spent outside, time spent cycling and exercising, whether it was a working day or a free day, and in case of a working day, what hours they worked. From the actigraph data, time spent in physical activity of different intensities and sedentary time will be derived based on accelerometer cut–off points in counts per minute. To measure sedentary behavior, the sufficiently valid and reliable adapted Workforce Sitting Questionnaire (WSQ) will also be used [59].

#### Diet behaviors

To gain more insight into the diet behaviors of night–shift workers and non–night–shift workers, food diaries will be used. Participants will be asked to keep a food diary for 3 consecutive days [30]. In the food diary, participants can report the time of the day at which the food is consumed and the type and amount of food that is consumed. The eating episodes of the participants will be categorized by means of the Food–Based Classification of Eating Episodes (FBCE) [60]. This instrument was specially developed to compare meal patterns and meal balance between night–shift workers and non–night–shift workers and is regarded as a reliable concept for food classification [60]. The food diaries and the categorization of participants’ dietary patterns by means of the FBCE will be used to assess participants’ timing of nutrition, frequency of eating, and snacking behavior.

#### Light exposure

To objectively measure (sun) light exposure, a subsample of the study population will be asked to wear a UV–sensitive light sensor (HOBO Pendant Light Data Logger) for 7 consecutive days to record UV and light intensity. This subsample will consist of the same participants (*n* = 260) who will wear the actigraphy devices. The light sensor will provide data on light exposure in 10-min bins of light exposure above a threshold of 10 lumens/ft2. This data will be used to compare light exposure in 3 timeframes during 24-h (day, evening, night) between night–shift workers and non–night–shift workers.

#### Vitamin D level and immunological factors

Blood samples will be drawn from the 175 expected cases of ILI/ARI and 70 controls. Sterile coagulation tubes will be used for the analysis of serum biomarkers including cytokines (pro–inflammatory) and other biomarkers of inflammation (e.g. C–reactive protein) using luminex assay, and for the analysis of vitamin D levels. Furthermore, EDTA tubes will be used for the analysis of biomarkers such as cortisol, melatonin, insulin, free fatty acids, cholesterol, and metabolic hormones. Sterile heparin tubes will be used to analyze a set of specific cellular biomarkers including specific Thelper subsets (Th1, Th2, Treg, and Th17), activation markers and functional assays into cytokine responsiveness or proliferation. To this end, flow cytometry will be used. Lastly, to examine mRNA markers by transcriptomics (the study of RNA transcripts), blood samples will be collected using PAXGENE blood mRNA tubes [48].

#### Other study parameters

Other study parameters will involve variables that may play a (modifying) role in the relation between night–shift work and health. Previous studies have for example indicated that, in general, young individuals, males and evening types are better able to adapt to night–shift work without adverse consequences [2, 61, 62]. The following variables will be measured by self–report, based on existing validated questionnaires:

- Smoking (4 items on current and past smoking behavior) and alcohol use (7 items on current alcohol use behavior);
- Job satisfaction (1 item on the extent to which one is satisfied with his/her job [63–65]);
- Work–life balance (4 items from the Survey Work–home Interference NijmeGen (SWING) [66, 67]);
- Socio–demographic factors (6 items on age, gender, ethnicity, level of education, employment status, and marital status);
- Chronotype (1 item from the Munich ChronoType Questionnaire (MCTQ) on whether a person is a morning or evening type [68]);
- Sickness absenteeism and presenteeism (8 items from the Dutch version of the World Health Organization’s Heath and Work Performance Questionnaire (HPQ) on sickness absenteeism and overall job performance [69]).

Table 2 provides an overview of the study parameters and their measurement methods.

**Table 2:** Overview of the study parameters, measurement methods and instruments

Parameter	Specification	Measurement method	Instrument	Source
*Primary study parameters*				
(Night−) shift work^a^	−Shift system	Questionnaire	Nightingale study questionnaire	Pijpe et al. 2014
	−Cumulative exposure			
	−Shift intensity			
Body weight^a^	−Body height	Anthropometry	Direct body height, weight, and waist circumference measurements	
	−Body weight			
	−Waist circumference			
	−BMI			
Infection susceptibility	−Influenza like illness	Daily log (app)	Mobile phone application	
	−Acute respiratory infection			
*Secondary study parameters*				
Sleep factors^a^	−Sleep quantity	Questionnaire;	MOS Sleep Scale;	Hays et al. 2005
	−Sleep quality	Actigraphy	Actigraphy devices	
	−Sleeping pattern			
Physical activity^a^	−Duration	Questionnaire;	SQUASH;	Wendel−Vos et al. 2003; Chau et al. 2011
	−Frequency	Actigraphy	WSQ;	
	−Intensity		Actigraphy devices	
	−Sedentary behavior			
Diet behaviors^a^	−Timing of nutrition	Food diary	Food diary and FBCE	Lennernäs & Andersson 1999
	−Frequency of eating			
	−Snacking behavior			
Light exposure^a^	−Artificial light exposure	Light sensor	HOBO Pendant Light Data Logger	
	−Sun light exposure			
Vitamin D level^a^	−Vitamin D level	Blood sample analyses	25−hydroxyvitamin D analysis	
Immunological factors^a^	−mRNA	Blood sample analyses	Transcriptomics; Thelper subset and cytokine profile analysis	
	−Lymphocytes			
	−Cytokine profiles			
*Other study parameters*				
Socio–demographic factors^a^	−Age	Questionnaire	Dutch Public Health Monitor	GGD’en, CBS & RIVM, 2012
	−Gender			
	−Ethnicity			
	−Level of education			
	−Employment status			
	−Marital status			
Smoking^a^	−Smoking behavior	Questionnaire	Dutch Public Health Monitor	GGD’en, CBS & RIVM, 2012
Alcohol use^a^	−Alcohol use behavior	Questionnaire	Dutch Public Health Monitor	GGD’en, CBS & RIVM, 2012
Job satisfaction	−Job satisfaction	Questionnaire	TAS	Smulders et al. 2001
Work−life balance	−Work–life balance	Questionnaire	SWING	Wagena & Geurts, 2000
Chronotype^a^	−Morning/evening type	Questionnaire	MCTQ	Roenneberg et al. 2003
Sickness absenteeism	−Sickness absenteeism	Questionnaire	HPQ	Kessler et al. 2003
	−Presenteeism			

*BMI* body mass index, *CBS* statistics Netherlands, *FBCE* food–based classification of eating episodes, *GGD* community health service, *HPQ* heath and work performance questionnaire, *MCTQ* Munich chrono type questionnaire, *MOS Sleep Scale* medical outcomes study sleep scale, *RIVM* national institute for public health and the environment, *SQUASH* short questionnaire to asses health enhancing physical activity, *SWING* survey work–home interference nijmeGen, *TAS* TNO work situation survey, *WSQ* workforce sitting questionnaire

^a^Study parameters that are also included in the Klokwerk study

### Statistical analysis

Regression analyses will be used to determine the association between night–shift work and BMI as well as between night–shift work and infection susceptibility, adjusted for confounders. Logistic regression analyses will be conducted for dichotomous dependent variables and linear regression analyses will be used for continuous dependent variables. Multilevel analyses will be used to take into account within–subject correlation due to repeated measurements and clustering of observations of health care workers within the same hospital/department. P–values less than 0.05 will be considered statistically significant.

The mediating role of sleep, physical activity, diet behaviors, light exposure, vitamin D, and immunological factors in the relationship between night–shift work and BMI and infection susceptibility will be examined by mediation analysis techniques. The mediating effect will be analyzed by the product of coefficient approach consisting of three regression analyses [70], followed by a Sobel test to determine the significance of the mediating effect [71]. Analyses will be done separately per outcome and per mediating variable.

The steps to be taken are to conduct a:

1. Univariate regression analysis with the independent variable (night–shift work) predicting the outcome (BMI/infection susceptibility);
2. Univariate regression analysis with the independent variable (night–shift work) predicting the mediating variable (e.g. sleep);
3. Multiple regression analysis with independent variable (night–shift work) and mediating variable (e.g. sleep) predicting the outcome (BMI/infection susceptibility).

In case of significant relations in steps 1–2, step 3 will be performed, where (partial or full) mediation is confirmed if the effect of the mediating variable remains significant after controlling for night–shift work. Full mediation is concluded if the (significant) relation between night–shift work and BMI/infection susceptibility disappears after controlling for the mediating variable. Otherwise, there is partial mediation (i.e. both night–shift work and sleep predict BMI/infection susceptibility). To test the significance of the mediating effect, subsequently a product of coefficients approach (multiplying two regression coefficients) will be performed and a standard error of the mediated effect will be calculated using the Sobel test [71].

Analyses will be carried out using IBM SPSS Statistics, version 22.0 (New York: IBM Corp).

## Discussion

Night–shift work may cause severe disturbances in the worker’s circadian rhythm, which has been associated with the onset of health problems and diseases. As a substantial part of the workforce is exposed to night–shift work, harmful aspects of night–shift work may have a large societal impact and should not be overlooked. Although effort has been made to fill the knowledge gap, much remains unclear about the interrelations between night–shift work, psychosocial, behavioral, and physiological factors, and health (i.e. body weight and infection susceptibility). The Klokwerk + study is an observational study in which the effects of night–shift work on body weight and infection susceptibility and the mechanisms underlying these health effects are studied. Due to its prospective design, large sample size, and comprehensive approach in studying potential mechanistic factors, this study will help to address the current research gap regarding the relation between night–shift work and overweight and infectious diseases. Based on the findings of Klokwerk+, interventions that prevent negative health effects of night–shift work can be developed. For example, if the findings indicate that diet plays an important mechanistic role in the development of negative health outcomes of night–shift work, interventions could be developed that target this modifiable behaviors (e.g. advising to eat at particular times during a night–shift period). Furthermore, the identification of biomarkers for circadian disturbance associated with night–shift work may be an important asset in monitoring the effects of such interventions. These efforts could eventually contribute to the establishment of prevention initiatives for night–shift workers that may subsequently also lead to reduced health care costs and productivity loss costs.

Several issues as to the design and execution of Klokwerk + may influence the study findings and should therefore be taken into account. As in most other observational studies, multiple study parameters will be assessed based on self–reported information. However, validated instruments will be used to measure these parameters. Furthermore, a strength of this study is that for several parameters, such as physical activity and BMI, objective data will also be collected. With respect to the study population, health care workers from multiple occupational groups will be included. Although this adds to the representativeness of our study sample, it will increase variability within our study sample, which may reduce internal validity. Another issue is related to the definition of night–shift work. It was decided to follow the definition given by Pijpe et al. (2014) [51], i.e. night–shift work is defined as working night shifts for ≥1 night/month over the past 6 months. However, as different aspects of shift work will be taken into account, we will be able to study different levels of (night-) shift work intensity and duration. Lastly, the recruitment of non–night–shift workers may require additional effort, as this group of health care workers may be underrepresented in hospitals and they may be less concerned with the topic of interest (i.e. night–shift work). In order to ensure that there is an adequate representation of both night–shift workers and non–night–shift workers in the study population, the distribution of night–shift work exposure in the study population will be monitored midway through the recruitment period. If there is a largely unequal distribution of night–shift workers and non–night–shift workers, additional recruitment strategies will be used to recruit more night–shift workers or non–night–shift workers.

In conclusion, the Klokwerk + study will contribute to the current need for high–quality data on the health effects of night–shift work and its underlying behavioral and physiological mechanisms. This knowledge is pivotal in reducing the burden that night–shift work may impose on a large, and still rising, number of workers.

## Abbreviations

ARI, acute respiratory infection; BMI, body mass index; CBS, statistics Netherlands; ECDC, European center for disease prevention and control; FBCE, food–based classification of eating episodes; GGD, community health service; HPQ, heath and work performance questionnaire; ILI, influenza–like illness; IRAS, institute for risk assessment sciences; MCTQ, Munich ChronoType questionnaire; MOS Sleep, medical outcomes study sleep scale; NKI, Dutch cancer institute; RIVM, national institute for public health and the environment; SQUASH, Short QUestionnaire to ASses Health enhancing physical activity; SWING, survey work–home Interference NijmeGen; TAS, TNO work situation survey; UMCU, University Medical Center Utrecht; WHO, world health organization; WSQ, workforce sitting questionnaire
